# Epidemiological and Surgical Insights into Inverted Papillomas: A Tertiary Center Experience

**DOI:** 10.7759/cureus.86121

**Published:** 2025-06-16

**Authors:** Rezarta Taga Senirli, Özer Erdem Gür

**Affiliations:** 1 Otolaryngology - Head and Neck Surgery, Antalya Training and Research Hospital, Antalya, TUR; 2 ENT/Sinus Surgery, Antalya Training and Research Hospital, Antalya, TUR

**Keywords:** endoscopic surgery, inverted papilloma, malignant transformation, sinonasal tumors, surgical outcomes

## Abstract

Objective

This study aimed to evaluate the epidemiological characteristics, clinical presentation, diagnostic methods, and surgical outcomes of sinonasal inverted papillomas (IPs) and to compare these findings with those in the existing literature.

Methods

A retrospective review was performed involving 25 patients diagnosed with IPs and treated by a single surgeon at Antalya Education and Research Hospital, a tertiary care center, between January 2015 and December 2021. Data regarding demographics, presenting symptoms, imaging findings, and postoperative outcomes were analyzed.

Results

Of the 25 patients, 23 were male and two were female (male-to-female ratio: 11.5:1). The mean age of the cohort was 54.7 years. The most common symptom was nasal obstruction. The tumor most frequently originated from the medial wall of the maxilla (40%). Pure endoscopic resection was performed in 20 patients, while four (16%) required revision surgery. Malignant transformation occurred in two patients - one with microinvasive squamous cell carcinoma and another with carcinoma in situ.

Conclusions

Although histologically benign, inverted papillomas demonstrate locally aggressive behavior and carry a risk of malignant transformation. Endoscopic surgical techniques offer favorable outcomes with low recurrence rates, but long-term surveillance remains critical.

## Introduction

Inverted papillomas (IPs) were first described in 1854. These tumors primarily originate in the sinonasal region, making up the majority of Schneiderian papillomas [1]. They represent approximately 0.5-7% of all sinonasal tumors and typically affect middle-aged adults [2]. IP is a benign but locally aggressive epithelial tumor originating from the Schneiderian membrane lining the nasal cavity and paranasal sinuses. It is characterized by its high recurrence rate, local invasiveness, and potential for malignant transformation.

The WHO classifies IP under sinonasal papillomas, with three major histologic types: inverted (endophytic), exophytic (fungiform), and oncocytic (cylindrical cell). Of these, the inverted subtype is the most clinically significant due to its recurrence potential and association with squamous cell carcinoma. IPs are predominantly observed in male patients (male-to-female ratio of 2-3:1) [3]. Although histologically benign, IPs require careful monitoring due to their locally aggressive behavior, high recurrence rates, and potential for malignant transformation or concurrent malignancy at diagnosis. Clinically, patients with IPs often present with nonspecific symptoms such as unilateral nasal obstruction, nasal discharge, epistaxis, and loss of smell. Since these symptoms are commonly associated with other sinonasal conditions, diagnosis may be delayed. Furthermore, due to their resemblance to inflammatory nasal polyps on examination, diagnosis can be further postponed. Consequently, histopathological evaluation is particularly important for patients presenting with unilateral polyps. The exact etiology of IPs has remained uncertain since their initial description. While the role of Epstein-Barr Virus (EBV) has been investigated, no definitive conclusions have been drawn [1]. Some studies suggest a potential link to low-grade human papillomavirus (HPV) infections, particularly types 6 and 11, as well as to high-grade HPV infections [3]. Additionally, environmental factors such as chronic inflammation, exposure to industrial chemicals, and smoking are believed to contribute to the development of these tumors. Radiological imaging is essential for the diagnosis and management of IPs, as it enables precise localization and assessment of tumor spread. Paranasal sinus CT is commonly requested to determine tumor extent, with studies indicating that intralesional microcalcifications are present in approximately 20% of cases [4]. MRI is also frequently used alongside CT to enhance evaluation of disease spread and to help in surgical planning. Surgical excision is the primary treatment for IPs. While open surgeries such as lateral rhinotomy with medial maxillectomy were initially performed, most surgeons now favor endoscopic surgery since it was first described by Waitz and Wigand [5]. Advances in surgical techniques and a deeper understanding of IP molecular biology have further enhanced patient management and outcomes. In this study, we analyze the data drawn from Antalya Training and Research Hospital, a tertiary care center, on IPs, focusing on epidemiological characteristics and surgical outcomes. We conducted a retrospective review of patients diagnosed with IP between 2015 and 2021. We aim to provide an overview of the epidemiology, pathophysiology, clinical presentation, diagnostic strategies, and management options for IPs by comparing our own cases with other series in the literature. Importantly, our study provides region-specific data from the Mediterranean region of Turkey, helping to expand the global understanding of IPs with insights from this geographic context.

## Materials and methods

Ethical approval for the study was obtained from the institutional review board of Antalya Training and Research Hospital, and the requirement for informed consent was waived due to the retrospective design. This study involves a retrospective case series of 25 sinonasal IP patients treated by a single surgeon at a tertiary healthcare center between January 2015 and December 2021. The files of patients identified with diagnosis codes J33 and D14.0 were reviewed, including anamnesis, CT, MRI, surgical notes, and follow-up processes. Patients who were newly diagnosed, as well as those who had previously undergone surgery at other institutions and subsequently required revision surgery by our team, were included in the study. Patients with insufficient follow-up data, a previous history of sinonasal malignancy, or a lack of essential preoperative imaging (CT and/or MRI) were excluded from the study to ensure the reliability and uniformity of the clinical outcomes.

The presenting symptoms of all patients, age at the time of surgery, imaging (CT/MRI) findings, preoperative biopsy results, and type of surgery performed were evaluated. Tumors were classified into four groups based on the Krouse classification system [6], using clinical findings, preoperative CT, and, when available, MRI. In addition, operative reports were meticulously reviewed for each case. Surgical approaches were categorized as purely endoscopic, open, or combined procedures. The choice of surgical technique was primarily based on tumor localization and extent. Patients without maxillary sinus involvement were treated with a purely endoscopic approach. In cases where the tumor extended to the maxillary sinus, particularly the anterior or anteroinferior wall, which are difficult to access, an endoscopically combined approach incorporating the Denker procedure was employed. Furthermore, patients were stratified as either primary or revision cases based on their surgical history.

## Results

Between January 2015 and December 2021, 25 patients with IP underwent surgery performed by a single surgeon. Of these patients, 23 were male and two were female (male-to-female ratio: 11.5:1). The age range of the patients was from 23 to 71 years, with an average age of 54.7 years. The most common presenting complaint was nasal obstruction, although nasal discharge and facial pain were also among the initial symptoms.

All patients were operated on under general anesthesia. In 40% of the cases, the origin of the IPs was identified as the medial wall of the maxilla. Other identified sites included the ethmoid sinus (20%), lamina papyracea (8%), middle turbinate (12%), inferior turbinate (12%), floor of the maxillary sinus (4%), and lateral wall of the maxillary sinus (4%) (Table 1). Revision surgery was performed in four patients (16%). In one of the revision cases (4%), a combined approach was required to reach an adhesion located on the lateral wall of the maxillary sinus.

**Table 1:** IP origin regions IP: inverted papilloma

Tumor origin	Number of patients	Percentage of patients (%)
Medial wall of maxilla	10	40
Floor of maxillary sinus	1	4
Lateral wall of maxillary sinus	1	4
Ethmoid sinus	5	20
Lamina papyracea	2	8
Middle turbinate	3	12
Inferior turbinate	3	12

**Figure 1:**
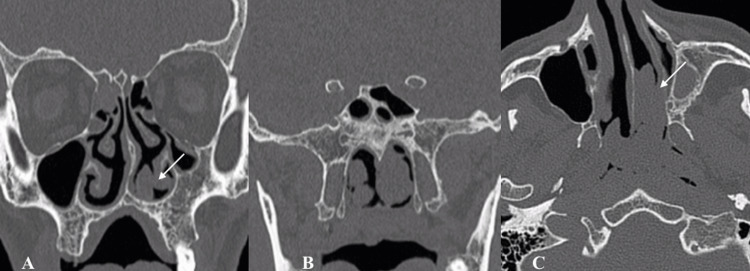
Inferior turbinate IP CT sections showing an IP (indicated with an arrow) in a 23-year-old male patient. A: Coronal CT scan showing a soft tissue mass originating from the left inferior turbinate, associated with bony remodeling (arrow). B: Posterior coronal CT section demonstrating ethmoid sinus involvement with adjacent bone thinning. C: Axial CT section of the same mass, with evidence of bone remodeling without aggressive destruction (arrow) CT: computed tomography; IP: inverted papilloma

**Figure 2:**
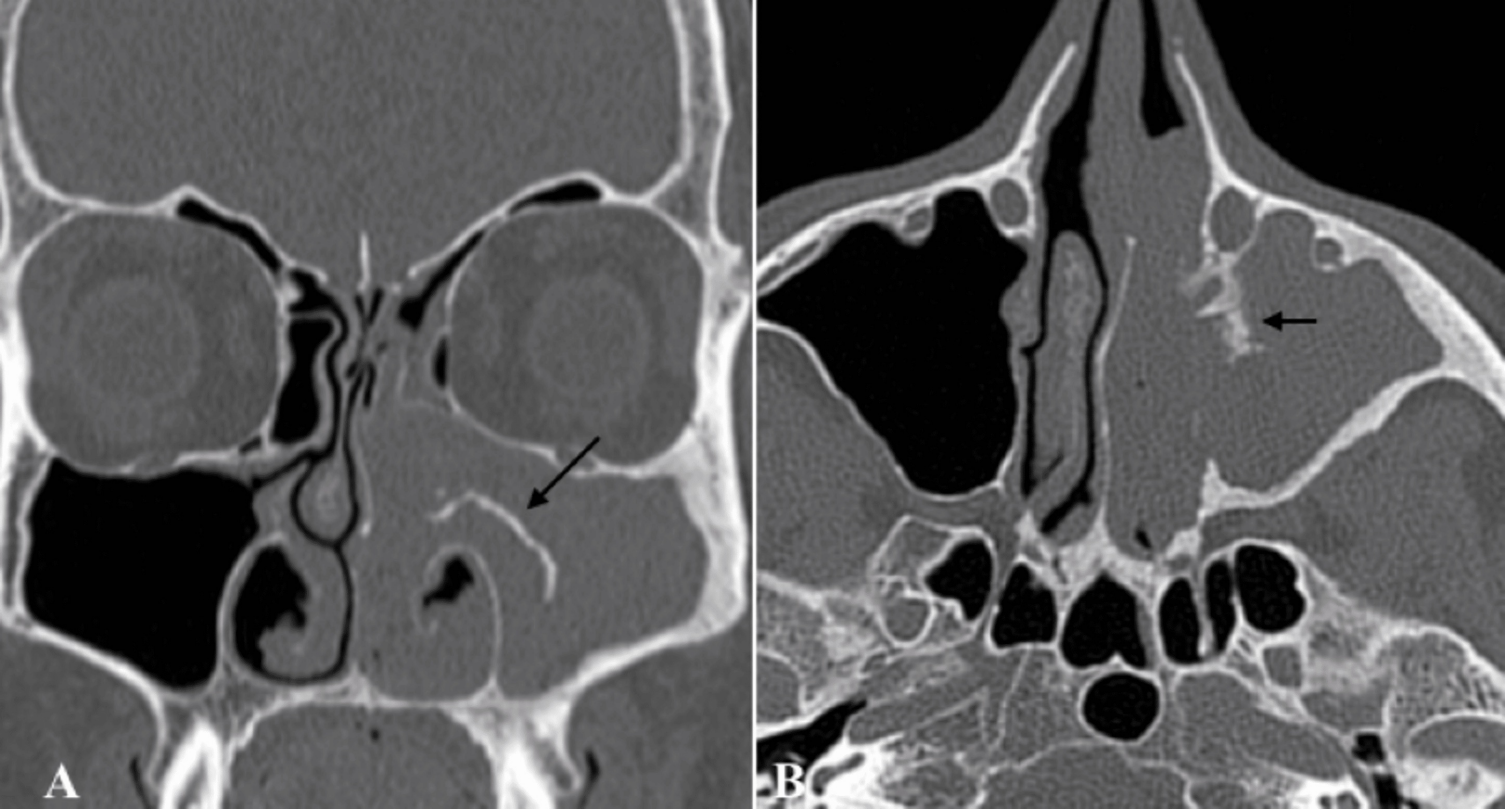
Maxilla medial wall IP CT sections (coronal and axial, respectively) showing an IP, indicated with an arrow, originating from the medial wall of the left maxilla CT: computed tomography; IP: inverted papilloma

**Figure 3:**
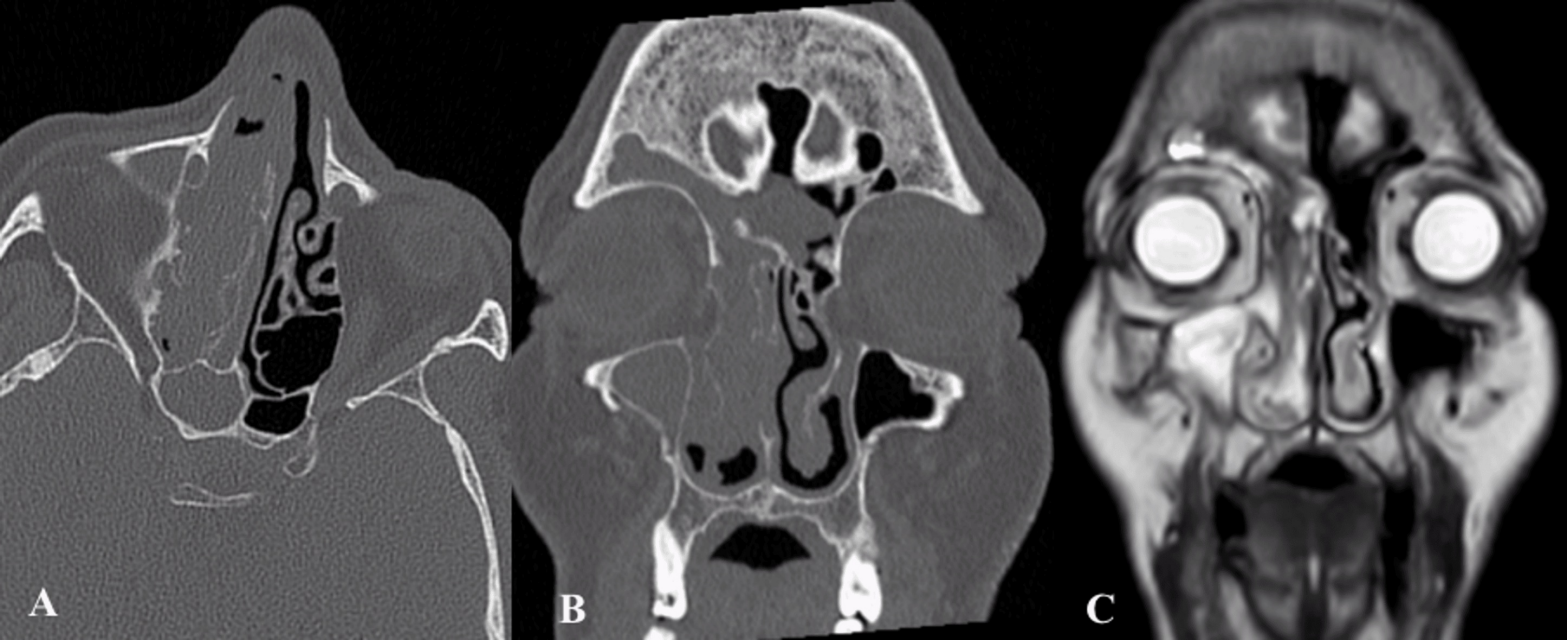
T4 IP Axial and coronal sections CT (A, B) and coronal section MRI (C) showing a T4 IP CT: computed tomography; IP: inverted papilloma; MRI: magnetic resonance imaging

According to the Krouse classification, 8% of the patients were categorized as having T1, 32% as T2, 52% as T3, and 8% as T4 IP. A combined surgical approach was used in five (20%) of these patients, while the rest were treated with an endoscopic method. The most commonly performed surgical interventions were medial maxillectomy and ethmoidectomy. In three patients, a Draf II/III procedure was deemed necessary, while a modified Denker procedure was employed in seven patients. The imaging sections of some demonstrative IPs originating from the inferior turbinate, the medial wall of the maxilla, and a widespread T4 case operated on by us are shown in Figures 1-3.

In 23 patients, the diagnosis was reported as IP, while microinvasive squamous cell carcinoma (SCC) was identified in one patient, and carcinoma in situ areas were found in another. The average follow-up period was 33 months. Minor complications such as facial pain and epistaxis were observed in the early postoperative period, while in the late postoperative period, a mucocele developed in one patient. No recurrences or lacrimal system complications were encountered in any of the patients.

## Discussion

IPs are benign sinonasal tumors known for their locally aggressive behavior, high recurrence rates, and potential for malignant transformation. Due to these characteristics, it is essential to perform an in-office punch biopsy to establish a preliminary diagnosis of the condition, allowing for more extensive surgery to prevent recurrence. IPs are observed more frequently in males, with a male-to-female ratio of 2-3:1 [7]. In our study, this ratio was significantly higher (11.5:1). Factors such as occupation, lifestyle, smoking, or industrial exposure could potentially contribute to this elevated ratio. In this study, it was determined that 60% of patients hailed from the Mediterranean region and 40% from the Central Anatolian region. IPs are predominantly seen in adulthood, with an average age at diagnosis of 55 years [8]. In our study, the age distribution aligns with the literature, indicating that IPs affect middle-aged and older adults, with an average age of 54.7 years. The most common presenting symptoms of IPs, such as nasal obstruction and nasal discharge, are nonspecific and can often be mistaken for more common sinonasal conditions [8,9]. This can lead to delays in diagnosis. As observed in our study, considering IPs in middle-aged males presenting with unilateral nasal symptoms is crucial for early diagnosis and treatment. Radiological imaging also plays a pivotal role in the diagnosis and therapeutic evaluation in the management of sinonasal IPs. The integration of imaging findings with clinical and endoscopic outcomes aids in disease stage assessment. Coronally reformatted CT can provide the three-dimensional classification of the IP and can help with endoscopic surgery planning. IP on CT evaluation usually appears as a lobulated soft tissue mass with or without calcification. Unlike a malignant tumor, IP causes bone remodelling (thinning and bowing) and resorption rather than osseous destruction [10]. On the other hand, MRI evaluations of about 80% of IPs show a “convoluted cerebriform pattern”. This pattern has also been reported in malignant masses [11]. However convoluted pattern coupled with CT findings is highly suggestive of IP [10]. The surgical management of IPs has evolved significantly with advancements in endoscopic techniques. Recent studies have shown that cases treated with endoscopic surgery have better recurrence outcomes compared to open surgical cases [12]. In our study, most patients were treated entirely with endoscopic methods, with the Denker procedure added for more controlled resection of tumors on the anterior wall of the maxillary sinus. The endoscopic Denker approach is a technique that enables endonasal anterior maxillectomy without requiring a sublabial incision [13]. Revision surgery was performed on four patients (16%) whose initial surgeries had been conducted in other centers. During the follow-up period, recurrence was observed in one patient (4%). Similar to a recent meta-analysis [14], our study also emphasizes that previous surgeries are a significant risk factor for recurrent IP. The low recurrence rate in our study may be attributed to meticulous surgical techniques and the use of imaging studies for precise tumor mapping. We also believe that specialization in specific fields by surgeons is also important. Our lead surgeon in this study specializes in sinonasal surgery. The Krouse classification system has been a valuable tool for classifying disease extent in our patients. The majority of our cases (52 %) were classified as stage T3, which indicates extension into the paranasal sinuses outside the nasal cavity. This stage generally carries a higher risk of recurrence, underscoring the importance of comprehensive surgical excision. However, as noted by Yu et al. [15], it is essential to remember that the Krouse classification primarily addresses the extent of tumor bulk. The rate of malignant transformation in IPs is between 5 and 15% [2,8,15]. In our cohort, malignancy was detected in two cases, with pathology results reported as microinvasive SCC and carcinoma in situ. These findings highlight the importance of careful histopathological evaluation of all IP samples, especially in cases where dysplasia or carcinoma is present. This emphasizes the importance of early detection and aggressive treatment to reduce malignancy-associated risks (15). Although IPs are considered benign tumors, their clinical behavior necessitates close monitoring, particularly in the postoperative period. In our cohort, the average follow-up period was 33 months, highlighting the vital importance of long-term follow-up for comprehensive disease management.

Limitations

This study has several limitations. Firstly, it was a retrospective single-center study with a relatively small sample size, which may limit the generalizability of the findings. One contributing factor to the limited sample size and variability in follow-up is that a significant portion of the study period coincided with the coronavirus disease 2019 (COVID-19) pandemic, which disrupted routine clinical care and follow-up schedules. Furthermore, all surgeries were performed by a single surgeon, which, while ensuring consistency in technique, may introduce surgeon-related bias. Finally, although the average follow-up period was 33 months, longer surveillance would be necessary to fully assess the long-term recurrence and malignant transformation rates of IPs.

## Conclusions

IP is an uncommon benign sinonasal tumor with distinct epidemiological features and clinical behavior. Our tertiary center experience reinforces that IP predominantly affects middle-aged men and can be associated with significant morbidity due to recurrence and potential malignant transformation. Endoscopic surgical resection, with judicious use of combined approaches for extensive disease, offers excellent disease control with low complication rates. Careful long-term follow-up is critical for managing IPs, given the possibility of recurrence or delayed malignant change. Ongoing research into the molecular pathogenesis of IP, including the role of HPV and other factors, may further clarify which patients are at higher risk for aggressive behavior. Meanwhile, adherence to meticulous surgical technique and appropriate surveillance remains the cornerstone of successful management of IPs.
